# 

**Published:** 1878-09

**Authors:** 


*CONTENTS.*

| | PAGE | | PAGE |
|---|---|---|---|
| Autumnal Diseases | 571 | Spitting Blood | 588 |
| Washing | 573 | Small Bed Chambers | 589 |
| Feeding the Sick | 573 | Each the Best | 590 |
| Cold in the Head | 575 | Wearing Flannel | 592 |
| Bible and Business | 576 | The Four Wives' Portraits | 592 |
| The Best Summer Beverage | 583 | Be Careful | 596 |
| Is Consumption Contageous? | 584 | Eight to Sixteen | 597 |
| Table Manners | 585 | Drinking Water | 598 |
| Tomatoes | 587 | | |

